# Heterozygosity for *Pten* Promotes Tumorigenesis in a Mouse Model of Medulloblastoma

**DOI:** 10.1371/journal.pone.0010849

**Published:** 2010-05-26

**Authors:** Robert C. Castellino, Benjamin G. Barwick, Matthew Schniederjan, Meghan C. Buss, Oren Becher, Dolores Hambardzumyan, Tobey J. MacDonald, Daniel J. Brat, Donald L. Durden

**Affiliations:** 1 Department of Pediatrics, Aflac Cancer Center and Blood Disorders Service, Children's Healthcare of Atlanta, Emory University School of Medicine, Atlanta, Georgia, United States of America; 2 Winship Cancer Institute, Emory University School of Medicine, Atlanta, Georgia, United States of America; 3 Pathology and Laboratory Medicine, Emory University School of Medicine, Atlanta, Georgia, United States of America; 4 Department of Pediatrics, Memorial Sloan-Kettering Cancer Center, New York, New York, United States of America; 5 Department of Neurosurgery, Memorial Sloan-Kettering Cancer Center, New York, New York, United States of America; 6 Moores Cancer Center, University of California San Diego, La Jolla, California, United States of America; University of Texas M. D. Anderson Cancer Center, United States of America

## Abstract

**Background:**

Recent publications have described an important role for cross talk between PI-3 kinase and sonic hedgehog signaling pathways in the pathogenesis of medulloblastoma.

**Methodology/Principal Findings:**

We crossed mice with constitutive activation of *Smoothened*, *SmoA1*, with *Pten* deficient mice. Both constitutive and conditional *Pten* deficiency doubled the incidence of mice with symptoms of medulloblastoma and resulted in decreased survival. Analysis revealed a clear separation of gene signatures, with up-regulation of genes in the PI-3 kinase signaling pathway, including downstream activation of angiogenesis in *SmoA1*+/−; *Pten* +/− medulloblastomas. Western blotting and immunohistochemistry confirmed reduced or absent Pten, Akt activation, and increased angiogenesis in *Pten* deficient tumors. Down-regulated genes included genes in the *sonic hedgehog* pathway and tumor suppressor genes. *SmoA1*+/−; *Pten* +/+ medulloblastomas appeared classic in histology with increased proliferation and diffuse staining for apoptosis. In contrast, *Pten* deficient tumors exhibited extensive nodularity with neuronal differentiation separated by focal areas of intense staining for proliferation and virtually absent apoptosis. Examination of human medulloblastomas revealed low to absent PTEN expression in over half of the tumors. Kaplan-Meier analysis confirmed worse overall survival in patients whose tumor exhibited low to absent PTEN expression.

**Conclusions/Significance:**

This suggests that PTEN expression is a marker of favorable prognosis and mouse models with activation of PI-3 kinase pathways may be important tools for preclinical evaluation of promising agents for the treatment of medulloblastoma.

## Introduction

Medulloblastoma is the most common malignant brain tumor of childhood. Multimodality treatment with surgery, radiation, and chemotherapy cures many patients, but often leaves survivors devastated with long-term toxicities that affect their neurocognitive and growth potential. Despite clinical advances, up to 30% of children with medulloblastoma experience tumor progression or recurrence, for which no curative therapy exists. The lack of more effective, less toxic therapies stems from our imperfect understanding of medulloblastoma tumor biology.

Currently, patients diagnosed with medulloblastoma are treated based upon disease stage, age at diagnosis, and extent of resection using a combination of surgery, chemotherapy, and ionizing radiation (IR) [1]. The importance of histology in tumor biology and treatment responsiveness has been controversial. The World Health Organization (WHO) currently recognizes at least 5 subtypes of medulloblastoma: classic, desmoplastic, extensive nodularity (MBEN), large-cell, and anaplastic [2]. While large-cell and anaplastic medulloblastomas tend to behave more aggressively and desmoplastic and MBEN tumors tend to be associated with a better prognosis for survival, medulloblastomas often contain cells of more than one histology [3], [4]. In addition, factors such as age and disease stage have been associated with worse prognosis independent of histology and treatment protocols do not currently stratify patients based on tumor histology.

In an effort to improve treatment, tumors have also been classified based upon their cytogenetic and gene expression profiles [5], [6]. Losses on chromosome 17q, the most common cytogenetic abnormality in human medulloblastoma, have been associated with classic or large-cell histology, while losses on 9q have been associated with desmoplastic tumors. Cytogenetic analyses have also identified frequent allelic loss of chromosome 10q23.31, the locus of phosphatase and tensin homolog, *PTEN*, in human medulloblastomas [7]. PTEN dephosphorylates phosphatidylinositol-3,4,5-triphosphate (PIP_3_), and is a major inhibitor of signaling through the phosphatidylinositol 3-kinase (PI-3 kinase) pathway. Activation of PI-3 kinase signaling is a major driving force in progression of a majority of human neoplasms, including brain tumors [8]. Studies of human medulloblastoma have reported decreased expression of *PTEN* mRNA and protein, compared to normal cerebellum controls. In addition, the *PTEN* promoter has been found to be hypermethylated in 5 of 10 human cases of medulloblastoma. And, Immunohistochemistry (IHC) has detected increased staining for activated AKT in human medulloblastoma tissues, consistent with loss of upstream inhibition by *PTEN* [9]. We hypothesized that increased signaling through PI-3 kinase may influence medulloblastoma tumorigenesis in a mouse model.

We used the *SmoA1* transgenic mouse model of medulloblastoma [10] to study the effect of *Pten* loss on medulloblastoma tumorigenesis. We found that heterozygosity for *Pten*, in the context of constitutive overexpression of *Smoothened*, *SmoA1*, altered tumor histology and accelerated medulloblastoma tumorigenesis in *SmoA1* +/−; *Pten* +/− mice. Analysis by gene expression microarray revealed a clear separation of gene signatures, with downstream activation of angiogenesis and down-regulation of genes involved in cell cycle regulation in *SmoA1*+/−; *Pten* +/− medulloblastomas. Western blotting and IHC confirmed PI-3 kinase pathway activation and increased angiogenesis in *Pten* deficient tumors. Compared to tumors from control mice, *SmoA1*+/−; *Pten* +/− tumors exhibited extensive nodularity with neuronal differentiation separated by focal areas of intense staining for proliferation and virtually absent apoptosis.

Examination of human medulloblastoma tissue microarrays revealed a significant association between PTEN loss and poor survival. PTEN expression was low to absent in over half of human medulloblastomas. The majority of those dead of disease had low to absent PTEN expression. Kaplan-Meier analysis confirmed worse overall survival in patients whose tumor exhibited low to absent PTEN protein expression, suggesting that PTEN expression is an important marker of prognosis in medulloblastoma.

## Results

### Heterozygosity for *Pten* promotes tumorigenesis in a mouse model of medulloblastoma

**Figure 1:**
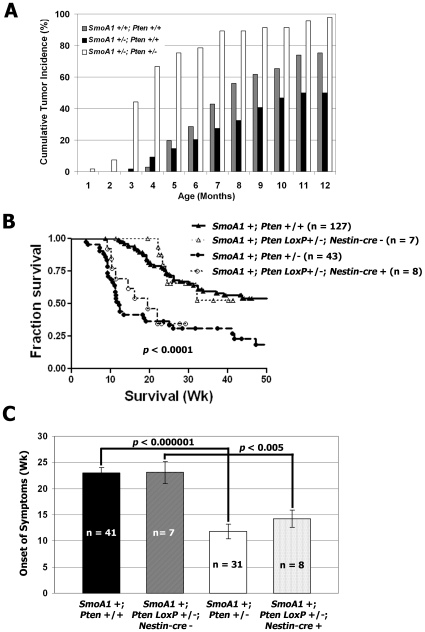
Compound *SmoA1*, *Pten* heterozygotic mice exhibit an increased incidence of medulloblastoma and reduced survival. *SmoA1* +/+ mice were crossed with *Pten* +/− mice and followed for symptoms. (**A**) Observed tumor incidence in *Pten* deficient versus control mice over the course of 1 year. (**B**) Kaplan-Meier survival analysis of *SmoA1* +; *Pten* +/− (n = 43) and *SmoA1* +; *Pten* (*LoxP*/*-*); *Nestin*-*cre* + (n = 8) mice, compared to *SmoA1* +; *Pten* +/+ (n = 127) and *SmoA1* +; *Pten* (*LoxP*/*-*); *Nestin*-*cre* - (n = 7) (p<0.0001, Log-rank) controls. (**C**) Mice with global *Pten* deficiency as well as those with conditional, partial knock-out of *Pten* developed symptoms earlier than controls (p<0.000001 and <0.005, respectively).

Approximately 50% of mice expressing one allele of *SmoA1*, *SmoA1* +/−, and 75% of mice expressing two alleles of *SmoA1*, *SmoA1* +/+, developed symptoms of medulloblastoma by 1 year of age (**Figure 1A**, **Table S1**). When *SmoA1* mice were crossed with *Pten* +/− mice, 97.9% of the *SmoA1* +/−; *Pten* +/− offspring followed over the same time interval exhibited symptoms of medulloblastoma. All symptomatic animals were necropsied and found to have visible tumor in the posterior fossa and tumor histology consistent with medulloblastoma. *SmoA1* +/−; *Pten* +/− mice exhibited decreased survival compared either to *SmoA1* +/+; *Pten* +/+ or to *SmoA1* +/−; *Pten* +/+ mice (*p*<0.0001, Log-rank) (**Figure 1B**).

We confirmed this finding using mice that express the *SmoA1* transgene, *SmoA1* +, and conditional, partial deletion of *Pten*, *Pten* (*LoxP*/-), in cells of neuronal lineage. In these triple transgene-positive mice, *Cre* was expressed under control of the *Nestin* gene promoter, *Nestin-cre*. We have not yet been able to generate mice that have complete deletion of *Pten* in *Nestin*-positive cells. However, we have evidence that conditional, partial knock-out of *Pten*, *SmoA1* +; *Pten* (*LoxP*/*-*); *Nestin*-*cre* +, accelerates medulloblastoma formation compared to controls, *SmoA1* +; *Pten* (*LoxP*/*-*); *Nestin*-*cre* - (**Figure 1B**). Mice with global *Pten* deficiency developed symptoms of medulloblastoma at a median age of 11.8±1.4 weeks and mice with conditional, partial knock-out of *Pten* developed symptoms at a median age of 13±1.7 weeks. In comparison, control *SmoA1* +; *Pten* +/+ and *SmoA1* +; *Pten* (*LoxP*/*-*); *Nestin*-*cre*- mice developed symptoms at median ages of 23±1 and 23.4±2.1weeks, respectively (**Figure 1C**).

### Loss of a single allele for *Pten* drives medulloblastoma histology to extensive nodularity

Mice that expressed the *SmoA1* transgene and that were either wild-type (+/+) or deficient (+/−) in expression of *Pten* were prone to development of tumor in the developing mouse cerebellum. Tumor was not identified in other areas of the mouse brain. The cerebellar tumors in this study varied in histologic appearance by whether the animal was homozygous or heterozygous for *Pten*, with the histologies corresponding to patterns of human medulloblastoma. The tumors in the *SmoA1*+; *Pten* +/+ mice showed a uniform pattern of “small round blue cells” arranged in sheets with molding of the tumor cell nuclei against each other and numerous mitoses and karyorrhectic tumor nuclei, recapitulating the appearance of classic medulloblastomas in humans. In contrast, the tumors from *SmoA1*+; *Pten* +/− mice were biphasic, with areas that were histologically identical to the tumors from *SmoA1*+; *Pten* +/+ mice, but also with large nodular areas of increased neuronal differentiation. These nodular areas were less cellular and had round nuclei that were arranged in a streaming pattern over a background of neuronal fibrillarity, similar to the appearance of the nodules of medulloblastomas with extensive nodularity (MBEN) in humans. Only rare mitoses and karyorrhectic nuclei could be identified in nodular areas (**Table S2**). This finding was confirmed in animals with conditional, partial knock-out of *Pten*, *SmoA1* +; *Pten* (*LoxP*/*-*); *Nestin*-*cre* +, which developed medulloblastomas with MBEN histology. Control *SmoA1* +; *Pten* (*LoxP*/*-*); *Nestin*-*cre* - animals developed medulloblastomas with classic histology (Data not shown).

### *SmoA1* +; *Pten* +/− medulloblastomas exhibit increased signaling through PI-3 kinase pathways

**Figure 2:**
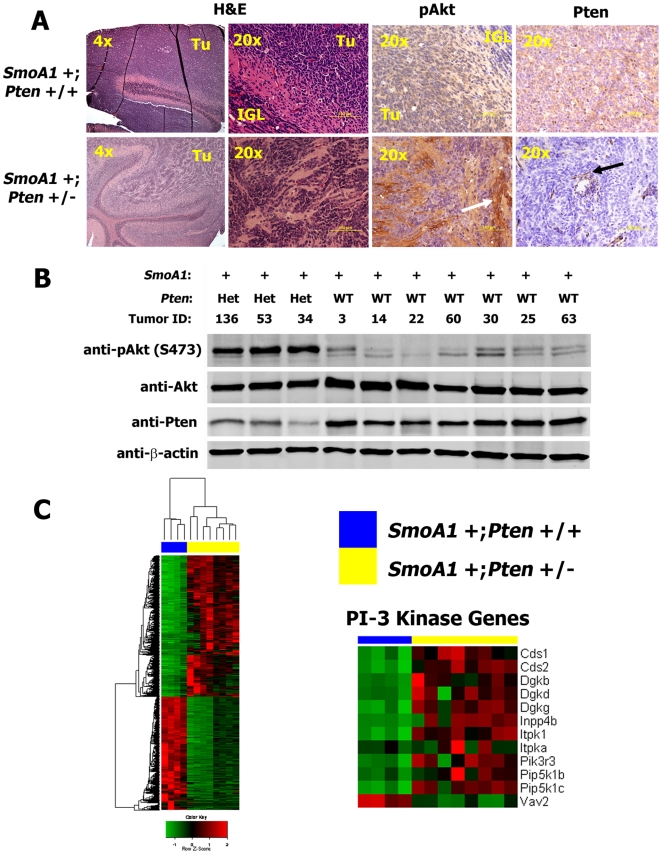
*Pten* deficiency activates PI-3 kinase signaling and drives medulloblastoma histology from classic to extensive nodularity. (**A**) *SmoA1* +; *Pten* +/+ medulloblastomas demonstrated classic histology; *SmoA1* +; *Pten* +/− tumors were extensively nodular in histology. *Pten* deficient tumors exhibited focal regions of intense staining for activated Akt (white arrow) and virtually absent staining for Pten. Only regions around blood vessels (black arrow) stained positive for Pten in *SmoA1* +/−; *Pten* +/− tumors. (**B**) Western blotting revealed decreased expression of Pten and increased activation of Akt in *SmoA1* +/−; *Pten* +/− medulloblastomas (n = 3), compared to controls (n = 7). (**C**) Total RNA was extracted from *SmoA1* +/−; *Pten* +/+ (n = 4) and *SmoA1* +/−; *Pten* +/− (n = 8) medulloblastomas and hybridized to Illumina mouse microarray chips. Expression of genes involved in PI-3 kinase signaling was up-regulated in *Pten* deficient tumors. Red pixels represent increased expression and green pixels represent decreased expression. Each column corresponds to mRNA extracted from an individual mouse medulloblastoma. The blue bar at the top of each heatmap indicates *Pten* +/+ and the yellow bar indicates *Pten* +/− tumors.

*SmoA1* +; *Pten* +/+ mouse medulloblastomas exhibited diffuse expression of Pten in tumor cells. In contrast, Pten expression was virtually absent in medulloblastomas from *SmoA1* +; *Pten* +/− mice, except in the areas around blood vessels (**Figure 2A** **, black arrow**). As expected, there was much stronger staining for activated Akt, phosphorylated on serine 473, in medulloblastoma tumors from *SmoA1* +; *Pten* +/− mice (**Figure 2A** **, white arrow**). By western blotting, we confirmed reduced Pten expression and increased expression of activated Akt, phosphorylated on serine 473, in *SmoA1* +; *Pten* +/− medulloblastomas (**Figure 2B**). Total Akt expression was equivalent among *SmoA1* +; *Pten* +/+ and *SmoA1* +; *Pten* +/− tumors.

To confirm our findings from IHC and western blotting, we extracted RNA from *SmoA1* +; *Pten* +/+ and *SmoA1* +; *Pten* +/− medulloblastomas, and hybridized labeled RNA to mouse Illumina microarray BeadChips for analysis of differential gene expression. Unsupervised hierarchical clustering revealed 2,724 probes that were differentially regulated between the two tumor types, including 1,511 probes that were overexpressed and 1,213 probes that were underexpressed in tumors from *SmoA1* +; *Pten* +/− mice (**Figure 2C, left panel**).

To assess the effects of *Pten* deficiency on molecular pathways that promote tumorigenesis, we used the KEGG (Kyoto Encyclopedia of Genes and Genomes) [11] and Gene Ontology [12] databases. *SmoA1* +; *Pten* +/− tumors expressed 1.1-fold less *Pten* mRNA than *SmoA1* +; *Pten* +/+ tumors. This result failed to achieve statistical significance, likely due to the low mRNA levels in *SmoA1* +; *Pten+/+* tumors. However, mRNA transcripts for components of the PI-3 kinase enzyme complex, *Pik3r3* and *Pik3cb*, and downstream targets of PI-3 kinase signaling, *Mapk8* (*Jnk*) and *Frap1* were up regulated 2.8, 1.9, 1.5, and 1.2-fold, respectively (**Figure 2C**,**Table S3**) in *Pten* deficient medulloblastomas.

The p55γ regulatory subunit of PI-3 kinase, *Pik3r3*, was recently found to be overexpressed in glioblastoma multiforme (GBM) brain tumors and has been implicated in promoting the growth of highly aggressive GBMs that lack amplification of the EGF receptor [13]. *Pik3cb* codes for the p110β catalytic subunit of PI-3 kinase and has been implicated as a driving force in *PTEN*-deficient tumors. Although activation of p110α (*PI3KCA*) is required to sustain the proliferation of established *PIK3CA*-mutant tumors, some *PTEN*-deficient tumors have been found to depend instead on signaling through p110β [14]. *Frap1* in mice codes for the mammalian target of rapamycin (mTOR), a known downstream target of PI-3 kinase. Development of prostate cancer in a mouse model with deletion of *Pten* specifically in prostate epithelium has been shown to require mTORC2, the mTOR complex 2 that contains the mTOR kinase and the Rictor regulatory protein [15].

### *Pten* +/− medulloblastomas down-regulate expression of targets of *sonic hedgehog* (*Shh*) signaling

**Figure 3:**
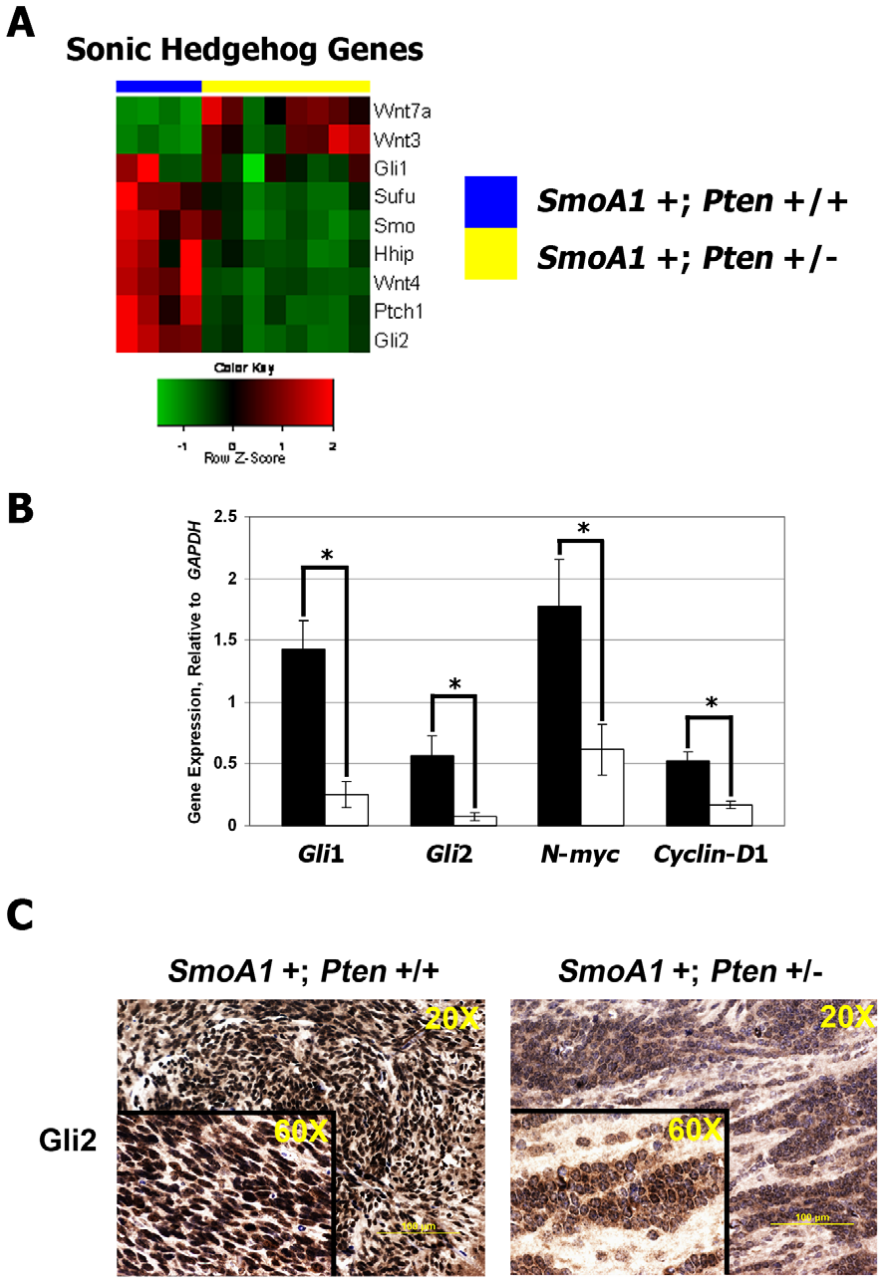
*SmoA1* +; *Pten* +/− medulloblastomas down-regulate expression of targets of *sonic hedgehog* (*Shh*) signaling. (**A**) Analysis of microarrays revealed that *Shh* pathway genes were down-regulated in *SmoA1* +; *Pten* +/− mouse tumors (n = 8) versus controls (n = 4). *Gli2*, a downstream target of *Smoothened*, was attenuated 4.2-fold. (**B**) Using real-time, RT-PCR we confirmed down-regulated expression of mRNA for downstream targets of *Smoothened*. Relative expression of *Gli1*, *Gli2*, *N-myc*, and *cyclin-D1* was decreased 5.6, 7.6, 4.3, and 2.8-fold (*, p<0.05 for all transcripts), respectively in *SmoA1* +; *Pten* +/− (white bars) medulloblastomas. *Error bars*, standard error of the mean. (**C**)We verified strong nuclear staining for Gli2, the major downstream target of activated *Shh* signaling, in *SmoA1* +; *Pten* +/+ medulloblastomas. Conversely, staining for Gli2 was both cytoplasmic and weaker in the nucleus in the nodules of *SmoA1* +; *Pten* +/− medulloblastomas.

Of interest is our finding that expression of genes involved in development of the cerebellum and in medulloblastoma tumorigenesis was decreased in *SmoA1* +; *Pten* +/− mouse tumors. Numerous studies have implicated *Shh* signaling in medulloblastoma pathogenesis, and modulation of this pathway has led to the vast majority of mouse models of medulloblastoma currently available. In addition, inactivating mutations of *PTCH1* and *SUFU* and activating mutations of *SMOH* account, in total, for at least 20% of all cases of medulloblastoma in humans [1]. In spite of the fact that both *Pten* +/− and *Pten* +/+ tumors expressed the *SmoA1* transgene by tail DNA genotyping, the expression of multiple genes in the *Shh-Smo* signaling pathway was down regulated in *Pten* +/− medulloblastomas (**Figure 3A**). Notably, multiple probes for *Gli2* and *Smo* were attenuated by 4.2, and 3.2-fold respectively (**Table S3**). Using real-time, RT-PCR we confirmed down-regulated expression of mRNA for downstream targets of *Smoothened*. Relative expression of *Gli1*, *Gli2*, *N-myc*, and *cyclin-D1* was decreased 5.6, 7.6, 4.3, and 2.8-fold (p<0.05 for all transcripts), respectively in *SmoA1* +; *Pten* +/− medulloblastomas (**Figure 3B**). IHC showed less intense staining for the *Shh*-signaling target Gli2 in nodules of *SmoA1* +;*Pten* +/− medulloblastomas (**Figure 3C**). In comparison, the genes *Wnt3* and *Wnt7a*, which were found to be up-regulated by gene expression microarray analysis, were validated as up-regulated by real-time, RT-PCR and western blotting in *SmoA1* +; *Pten* +/− medulloblastomas (**Figure S1**). Since Gli2 is well-recognized as the main transcriptional effector of *Shh* signaling in granule neuron precursor cells that form the cerebellum [16], this suggests that loss of *Pten* may actually attenuate signaling through *Shh* signaling pathways.

### *Pten* deficiency promotes neuronal differentiation in mouse medulloblastomas

Gene expression also revealed significant up-regulation of genes that promote neuronal differentiation and plasticity in *SmoA1* +; *Pten* +/− medulloblastomas. mRNA transcripts for *Bdnf* (*Brain-derived neurotrophic factor*), *Neurl* (*Neuralized homolog*), *Id4* (*Inhibitor of DNA binding 4*), and *Neurod2* (*Neurogenic differentiation 2*) were up-regulated 5, 3.7, 2.7, and 2.2-fold in *SmoA1* +; *Pten* +/− medulloblastomas (**Figure S2A**). Inactivation of Pten has been shown to promote *Bdnf*-mediated activation of Akt in cultures of rat primary neurons [17]. In addition, the transcription factor, *Neurod2*, is known to induce neuronal differentiation and promote survival of mature neurons [18].

To validate our findings from gene expression microarrays, we examined mouse medulloblastomas for markers of cell lineage using IHC. We observed intense staining for the marker of differentiated neurons, NeuN, in *Pten* deficient medulloblastomas and virtually no NeuN staining in *SmoA1* +; *Pten* +/+ tumors (**Figure S2B**). As a positive control, NeuN positive neurons were visualized in their expected location, in the internal granular layer (IGL) of the cerebellum, in both tumor types. Double-staining of tumor sections with an antibody against the proliferation marker PCNA and an anti-NeuN antibody revealed diffuse proliferation with scattered areas of staining for NeuN in *SmoA1* +; *Pten* +/+ medulloblastomas. *Pten* deficient tumors, in contrast, displayed larger islands of neuronal differentiation (**Figure S2C, brown staining**), surrounded by and distinct from PCNA positive (**Figure S2C, red staining**) areas of proliferation. Neither tumor type stained positive for the marker of astrocytic differentiation, GFAP (**Figure S2D**). Staining for synaptophysin, a marker of primitive neurons was weak in both tumor types, but appeared to overlap with staining for PCNA (**Figure S2E**). Thus, *Pten* deficiency appears to promote neuronal differentiation of medulloblastoma cells. The differentiated cells are juxtaposed with, but distinct from cells that are actively proliferating and that display features of immature neurons.

### *SmoA1* +; *Pten* +/− medulloblastomas exhibit up-regulation of angiogenesis

**Figure 4:**
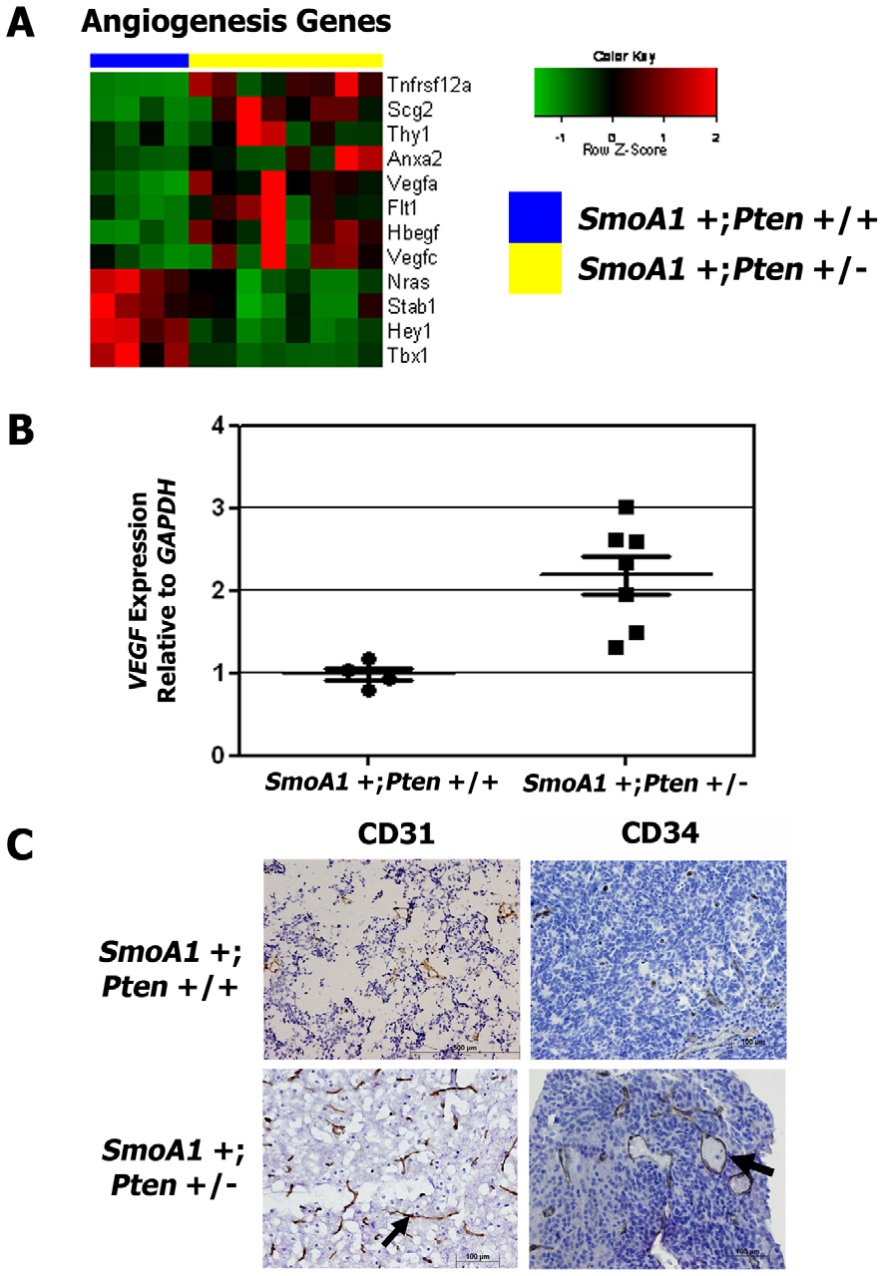
*Pten* +/− mouse medulloblastomas display increased angiogenesis. (**A**) *Pten* deficient medulloblastomas (n = 8) exhibited higher expression of genes involved in angiogenesis versus controls (n = 4). (**B**) Real-time, RT-PCR confirmed increased expression of *Vegfa* mRNA in *SmoA1* +; *Pten* +/− (n = 7) compared to *SmoA1* +; *Pten* +/+ (n = 4) tumors (*p* = 0.005). *Error bars*, standard error of the mean. (**C**) Increased CD31-positive staining of endothelial cells in *Pten* +/− (n = 4) (small black arrow), compared to *Pten* +/+ (n = 4) medulloblastomas. *SmoA1* +; *Pten* +/− tumors (n = 4) also exhibited increased CD34-positive staining and staining of larger-bore blood vessels (large black arrow) than *SmoA1* +; *Pten* +/+ medulloblastomas (n = 4).

Downstream of signaling through PI-3 kinase, PTEN has been shown to play a key role in regulating angiogenesis in brain tumors [8]. *Shh* signaling has also been described as crucial for angiogenesis during embryonic development [19]. However, the role of angiogenesis in human medulloblastomas has not been explored extensively. We identified up-regulated expression of numerous genes in *SmoA1* +; *Pten* +/− medulloblastomas that have been implicated in angiogenesis (**Figure 4A**). Notable genes include *Vegfa*, *Flt1*, and *Hbegf*, which were up regulated 2.3, 2.1, and 1.8-fold, respectively in *SmoA1* +; *Pten* +/− tumors (**Table S3**). Real-time, RT-PCR confirmed 2.4-fold increased median expression of *Vegfa* mRNA in medulloblastomas extracted from *Pten* deficient mice (n = 7), compared to controls (n = 4) (*p* = 0.005) (**Figure 4B**).

IHC of tumor tissue verified increased angiogenesis in *SmoA1* +; *Pten* +/− medulloblastomas. Staining of OCT-embedded tumor tissue with an antibody against the endothelial antigen CD31, revealed increased staining for CD31-positive blood vessels in *SmoA1* +; *Pten* +/− tumors (**Figure 4C** **, small black arrow**). We confirmed these findings by probing paraffin-embedded tissues for expression of the hematopoietic stem cell marker, CD34. Medulloblastomas from *SmoA1* +; *Pten* +/− mice exhibited increased staining for CD34 and evidence of larger-bore blood vessels (**Figure 4C** **, large black arrow**).

### *SmoA1* +; *Pten* +/− medulloblastomas exhibit increased proliferation and decreased apoptosis

**Figure 5:**
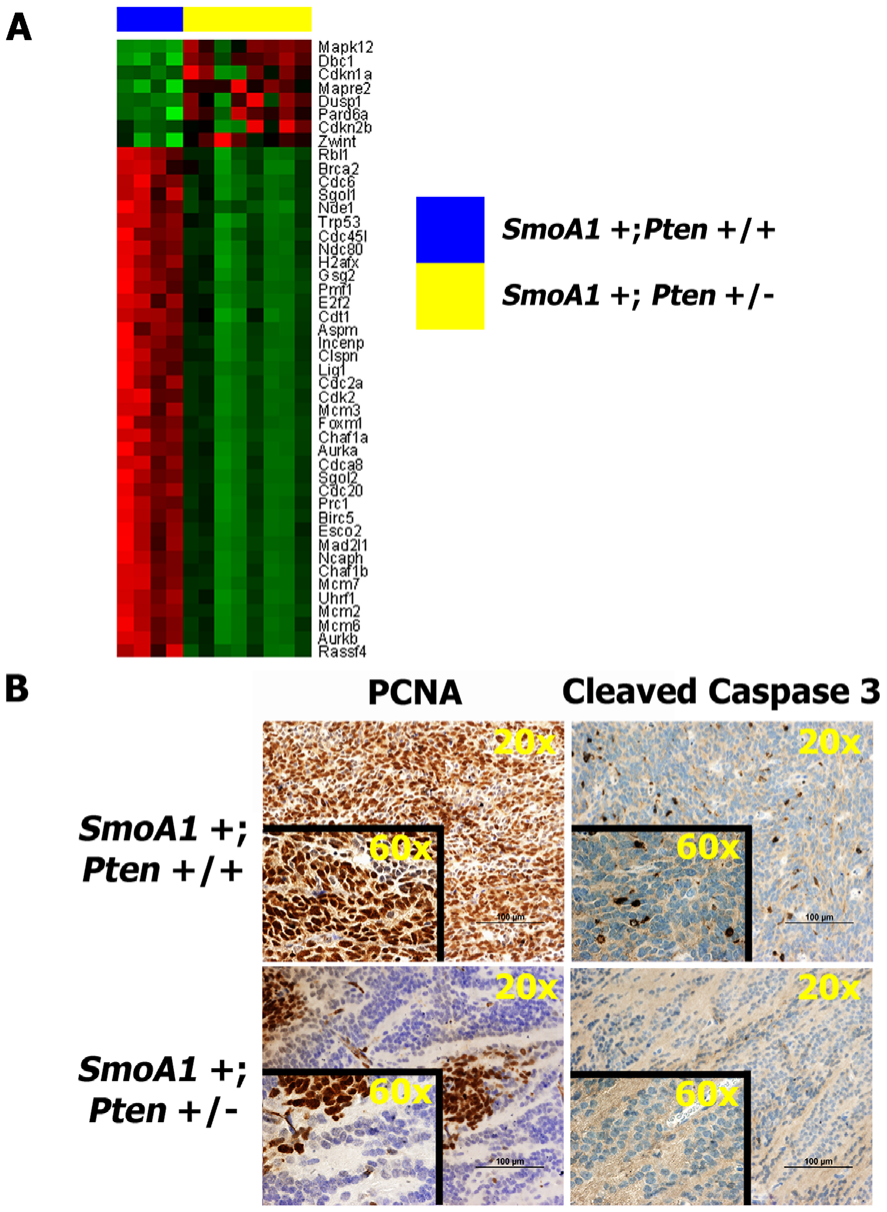
*Pten* +/− medulloblastomas exhibit down-regulation of genes involved in cell cycle control, with associated increased proliferation and reduced apoptosis. (**A**) Lower expression of genes involved in cell cycle control (**Table S3**) in tumors from *SmoA1* +/−; *Pten* +/− (n = 8) versus control (n = 4) mice. (**B**) Focal areas of intense staining for PCNA and virtually absent staining for Cleaved Caspase-3 in *Pten* +/− tumors (n = 5), compared to *Pten* +/+ medulloblastomas (n = 7).

Gene expression also revealed significant down-regulation of genes that control progression through the cell cycle in *SmoA1* +; *Pten* +/− medulloblastomas (**Figure 5A**). Expression of the key cell cycle regulators *Trp53*, *Brca2*, and *Rb1* was down regulated 2.5, 1.9, and 1.8-fold, respectively (**Table S3**). Western blotting confirmed overall lower expression of Trp53 in *SmoA1* +; *Pten* +/− mouse medulloblastomas (Data not shown).

IHC analysis identified islands of intense staining for the proliferation marker PCNA, surrounded by relatively quiescent areas in *Pten* deficient medulloblastomas. This pattern is similar to that seen in human medulloblastomas with extensive nodularity, where proliferation is limited to internodular tissue and is minimal within nodules of greater neuronal differentiation [20]. *SmoA1* +; *Pten* +/+ tumors displayed diffuse staining for PCNA throughout each tumor section. In contrast, staining for cleaved caspase 3, a marker of apoptosis, revealed diffuse staining throughout *SmoA1* +; *Pten* +/+ medulloblastomas, but virtually no expression in *SmoA1* +; *Pten* +/− tumors (**Figure 5B**). This suggests that decreased apoptosis is an important cause for our finding of increased mortality in *Pten* deficient mouse medulloblastomas.

### PTEN loss in human medulloblastoma is associated with a poor prognosis for survival

**Figure 6:**
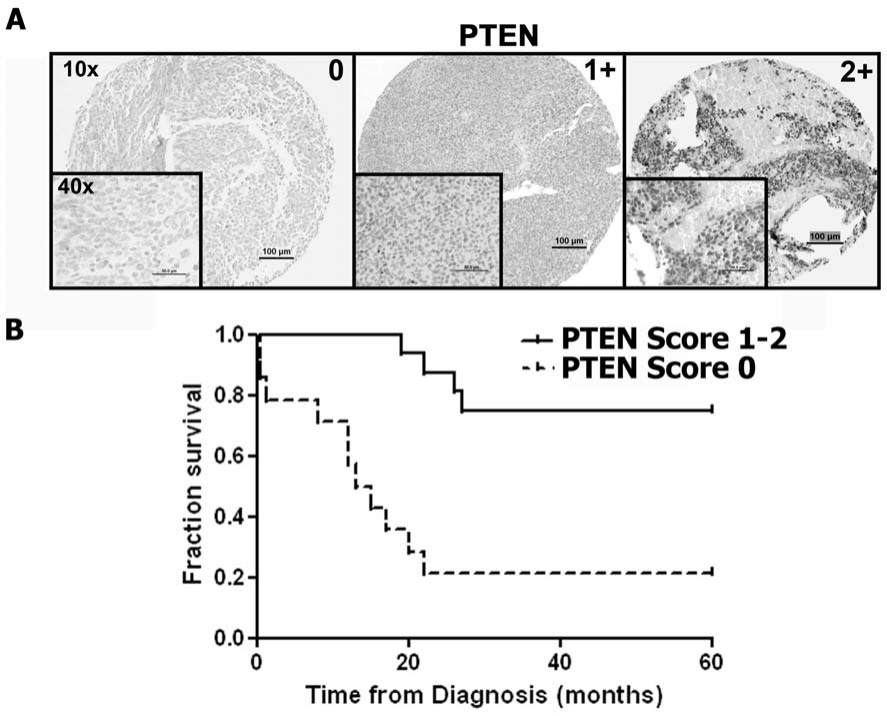
Loss of PTEN expression is an indicator of poor prognosis in patients with medulloblastoma. (**A**) PTEN expression was scored from 0 to 2+ in human medulloblastoma (n = 111) tumors. (**B**) Kaplan-Meier analysis confirmed worse overall survival in patients whose tumor exhibited low to absent (score = 0) PTEN expression (n = 16), compared to survival of patients with detectable PTEN (n = 26) expression (score = 1–2) (**Table 1**) by IHC (*p*<0.0005).

In order to validate our findings from mouse models, human TMAs were stained for expression of PTEN and were scored independently by two pathologists on a scale of 0 (no staining) to 2 (intense staining). By IHC, PTEN expression was low to absent (score = 0) in 61% of medulloblastomas (n = 111) (**Figure 6A** **,** **Table 1**). For patients known to be alive (n = 27) or dead (n = 15) with PTEN expression data available, of those alive, only 18% had low to absent PTEN expression by IHC. In contrast, 73% of those dead of disease had low to absent PTEN expression by IHC. Medulloblastomas from patients who died with PTEN deficiency (score = 0) were of either classic (n = 9) or desmoplastic (n = 2) histology and none had metastasis initially at diagnosis. Patients who died with detectable PTEN expression (score = 1–2) had anaplastic histology (n = 1) or had metastases at diagnosis (n = 3). Kaplan-Meier analysis confirmed worse overall survival in patients whose tumor exhibited low to absent PTEN protein expression compared to survival of patients with detectable expression of PTEN (*p*<0.0005) (**Figure 6B**), which suggests that loss of PTEN is an independent poor prognostic feature independent of tumor histology or disease stage.

**Table 1:** Absent PTEN expression is associated with increased mortality.

		<3 yo	3–18 yo	≥18yo	Unknown	TOTAL
**Classic Histology**	**PTEN 0**	1	4	7	10	22 (73%)
	**PTEN 1–2**	-	6	2	-	8
**Anaplastic Histology**	**PTEN 0**	2	3	2	14	21 (68%)
	**PTEN 1–2**	2	6	1	1	10
**Desmoplastic Histology**	**PTEN 0**	1	4	5	2	12 (54%)
	**PTEN 1–2**	-	7	3	-	10
**All Tumors**	**PTEN 0**	5	22	14	27	68 (61%)
	**PTEN 1–2**	4	30	7	2	43
**TOTAL**		9	52	21	29	111
**ALIVE**	**PTEN 0**	1	4	-	-	5 (18%)
	**PTEN 1–2**	1	20	1	-	22
**DEAD**	**PTEN 0**	3	5	3	-	11 (73%)
	**PTEN 1–2**	1	3	-	-	4

## Discussion

The ability to overcome mechanisms that promote apoptosis is essential for the development and progression of cancer. The PI-3 kinase signaling pathway provides such a mechanism by transmitting a strong survival signal. In humans, one of the major inhibitors of signaling through the PI-3 kinase pathway is PTEN. One of the first mouse models to examine potential cross-talk between *Shh* and PI-3 kinase signaling in medulloblastoma employed the *RCAS*/*tv*-*a* retroviral system for exogenous gene expression in the mouse neonatal cerebellum. Rao *et*. *al*. was the first group to report that co-overexpression of *Shh* and either activated *Akt* or *IGF2* significantly increased the incidence of medulloblastoma [21]. Another group demonstrated a high frequency of medulloblastomas in mice through co-overexpression of *Shh* and hepatocyte growth factor, *HGF*, the ligand for the transmembrane receptor *c-Met*. *HGF* overexpression activated PI-3 kinase signaling; and, compared to medulloblastomas generated by overexpression of *Shh* alone, exogenous *Shh* + *HGF* appeared to promote tumorigenesis by inhibiting apoptosis in granule neuron precursors of the developing cerebellum [22].

We found that *Pten* deficiency drove medulloblastoma tumors from classic to extensively nodular (MBEN) histology. MBEN tumors are characterized primarily by large nodules with a low proliferative index and neurocytic differentiation. MBEN tumors often contain smaller areas of proliferation, embedded in a dense network of reticullin fiber. Our finding of MBEN in *Pten* deficient tumors confirm those in prior reports in which tumors with extensive nodularity were generated through activation of PI-3 kinase signaling pathways. MBEN histology has been reported in medulloblastoma tumors generated when *Pten* was conditionally deleted in cells of neuronal lineage, using an RCAS/*tv*-*a* system [23]. Investigators have also generated a mouse model with MBEN by simultaneously overexpressing *Shh* and *HGF* [22]. Neither of these reports of mouse models with MBEN described an association of MBEN histology with decreased survival.

Our observation in mouse models is curious because it seems counter to the reported superior survival outcomes in children diagnosed with medulloblastoma with MBEN histology, compared to the survival of children with tumors of classic histology. In Gorlin syndrome, where patients have mutation of *PATCHED1* and thus activation of signaling through *Shh* pathways and an increased incidence of medulloblastoma, medulloblastoma tumors are usually desmoplastic or MBEN in histology [4]. Patients with desmoplastic or MBEN tumor histologies have historically had a much better survival prognosis than patients with classic medulloblastoma [2]. A recent retrospective analysis reported a 5-year survival of 92% in patients with medulloblastoma with MBEN histology, 90% with desmoplastic histology, and 66% in patients with classic medulloblastoma. MBEN medulloblastomas were associated with a lower risk of metastasis at diagnosis and increased sensitivity to chemotherapy and IR [4]. In contrast, *Shh*-driven tumors in mice may have some evidence of desmoplasia, but not true nodules, and overall look very classic in appearance [22], [24]. Yet, we have found that MBEN mouse medulloblastomas have a survival that is inferior to *Shh*-driven, classic mouse medulloblastomas. And, MBEN mouse medulloblastomas appeared to exhibit resistance to IR in one published report [23]. The significance of this disparity between tumor histology and survival in mouse models versus human medulloblastomas has yet to be addressed in the literature.

One mechanism that may explain the increased mortality of *Pten* deficient mice is increased angiogenesis. Persistent angiogenesis is one of the hallmarks that distinguish high-grade from low-grade brain tumors [25]. Our study found up-regulation of *Vegfa*, *Flt1*, and *Hbegf* in *SmoA1* +; *Pten* +/− medulloblastomas. Vascular endothelial growth factor A, *Vegfa,* and the receptor for *Vegfb*, *Flt1*(*Vegfr1*), are well described pro-angiogenic factors [26]. *Hbegf*, which codes for the heparin-binding EGF-like growth factor, has been established as a potent inducer of tumor growth and angiogenesis [27].

Tumor blood vessel density has also been identified as an independent prognostic factor in high-grade, malignant astrocytomas [28]. Early studies of human medulloblastomas identified increased microvascular density (MVD) in tumor tissue as compared to surrounding normal cerebellum [29]. However, there was high inter-tumor variability of MVD and MVD failed to correlate with metastatic status or patient survival [30]. A subsequent study found expression of a wide array of angiogenic factors including *VEGF165*, *PDGF-A*, and *VEGF-B* in 93% of human medulloblastomas [31]. We identified increased angiogenesis by IHC in *Pten* deficient medulloblastomas. This suggests that increased angiogenesis may be an important mechanism that negatively affects the survival of *Pten* deficient medulloblastomas.

Our study also identified down-regulation of cell cycle-related genes and significant reduction of apoptosis in *Pten* deficient medulloblastomas. Cell cycle genes *Trp53*, *Brca2*, and *Rb1* were down regulated in *Pten* +/− tumors. Loss of *RB1* or other components of the *RB1* pathway has been associated with decreased survival of patients with high-grade gliomas. This association was even stronger in combination with loss of wild-type *PTEN* [32]. Loss of *Brca2* has previously been linked to medulloblastoma tumorigenesis, as concomitant loss of *Trp53* leads to rapid formation of medulloblastomas in *Brca2*(*LoxP*/*LoxP*); *Nestin*-*cre* mice [33]. p53 is a well characterized downstream target of PI-3 kinase signaling. And, PTEN functions as a key inhibitor of PI-3 kinase signaling through its physical interactions with p53 to control cell proliferation [34].

Our findings are in agreement with the findings of McCall *et*. *al*. who reported decreased apoptosis in mouse medulloblastomas derived from co-overexpression of sonic hedgehog and activated Akt in the developing mouse brain [35]. Similarly, Binning *et al*. report in increased staining for the proliferation marker, Ki67, and decreased staining for cleaved caspase-3 in *Shh* + *HGF*-driven mouse medulloblastomas. Given that *SmoA1*+/−; *Pten* +/+ mice survive longer than *SmoA1*+/−; *Pten* +/− mice, it appears that haploinsufficiency for *Pten* drives differentiation of cells in the cerebellum toward a neuronal phenotype with associated regions of reduced apoptosis.

Importantly, we found that over one-half of human medulloblastomas in our study exhibited decreased expression of PTEN. Absence of PTEN expression was associated with increased mortality and reduced survival of patients. Only one other study has reported down-regulation of PI-3 kinase signaling in human medulloblastomas [9]. Using a larger cohort of patients, we detected absent PTEN staining in 61% of human medulloblastomas. Unlike the previous study, we show a significant difference in survival between patients whose tumor stains positively and negatively for PTEN expression. This suggests that not only does *Pten* deficiency promote tumorigenesis in our mouse model; but, PTEN deficiency may be a marker of poor prognosis in patients, independent of tumor histology or disease stage.

We believe that our mouse model is particularly attractive for pre-clinical drug development because of the ease with which *de novo* medulloblastoma tumors develop in *Pten* deficient mice and because of the difference in tumor histology between the two mouse models. Other models of activated PI-3 kinase signaling require expertise with neuro-surgery. We were able to achieve similar results by crossing *SmoA1* +/+ and *Pten* +/− mice, which are readily available. The *SmoA1* +/−; *Pten* +/− mouse model may be useful for examining the effects of anti-angiogenic therapies in MBEN versus classic medulloblastomas. The recent availability of small molecule inhibitors of signaling through PI-3 kinase pathways [36], many of which are in early Phase I/II trials in adult malignancies, also gives us an opportunity to examine the efficacy of targeted agents in a mouse model that closely recapitulates classic and nodular medulloblastomas, before these agents are tested on children with medulloblastoma.

## Materials and Methods

### Animal Husbandry

*Pten* +/− mice were a gift from Ramon Parsons (Columbia University, New York, NY). *ND2*:*SmoA1* (*SmoA1*) transgenic mice were a gift from James Olson (University of Washington, Seattle, WA). *SmoA1* +/+ founder genotypes were confirmed by fluorescent *in situ* hybridization (FISH) in the Olson laboratory. All offspring (n = 100) of *SmoA1*×*SmoA1* matings have tested positive for the *SmoA1* transgene and are thus considered homozygous for *SmoA1*, *SmoA1* +/+.

*SmoA1* +/+ mice were bred to *Pten* +/− mice to obtain *SmoA1* +; *Pten* +/+ and *SmoA1* +; *Pten* +/− F1 animals. All F1 offspring expressed the *SmoA1* transgene by tail PCR, and were considered to be hemizygous for the transgene, *SmoA1* +/−. *Pten LoxP*/*LoxP* (gift from W. David Martin, Emory University) were crossed with *SmoA1*+/+ mice. *SmoA1* +/+; *Pten LoxP*/*LoxP* offspring were crossed with *Nestin-cre* + (Jackson Laboratories, Bar Harbor, ME) mice to obtain *SmoA1* +; *Pten LoxP*/-; *Nestin-cre* + mice. Mice were observed for symptoms of medulloblastoma at least twice weekly for a period of 12 months. All mice were housed in an American Association of Laboratory Animal Care–accredited facility and were maintained in accordance with NIH guidelines. This was approved by the Institutional Animal Care and Use Committee of Emory University (Protocol # 145-2009). Survival was analyzed using GraphPad Prism 4 (GraphPad Software, Inc., La Jolla, CA).

### Mouse Necropsy and Tissue Handling

Mice were followed and sacrificed upon development of symptoms of medulloblastoma, which included head doming, hunched posture, preferential turning to one side, lethargy, and/or weight loss, using CO_2_ inhalation. The cerebellar tumor was either snap-frozen in liquid nitrogen for RNA studies, snap-frozen in Optimal Cutting Temperature (OCT) cryoembedding media (Tissue-Tek, Sakura Finetek, Torrance, CA), or fixed in 4% paraformaldehyde for pathological examination. OCT blocks were cut into 10–20-µm sections and stained with antibody [37]. Tissue blocks were paraffin embedded, cut into 4-µm sections, and then stained with hematoxylin and eosin (H&E) [38]. All necropsied brains were classified by a pathologist as medulloblastoma.

### Western Blotting and Immunohistochemical Analysis of Mouse Medulloblastomas

Proteins extracted from cells were electrophoretically separated on polyacrylamide denaturing gels, transferred onto nitrocellulose membranes, and immunoblotted with the designated antibodies as previously described [39]. Immunohistochemistry was performed according to manufacturer's recommendations. Antibodies used included Pten (Cell Signaling), phospho-Akt (Ser473) (Cell Signaling), p53 (DO-1) (Santa Cruz), Wnt-3 (Santa Cruz), Wnt-7 (Santa Cruz), β-actin (Sigma), NeuN (Millipore), GFAP (Abcam), Synaptophysin (Abcam), CD31 (BD Pharmingen), CD34 (BD Pharmingen), PCNA (Cell Signaling), and Cleaved Caspase 3 (Asp175) (Cell Signaling). Secondary antibodies were applied according to manufacturer's recommendations (Vector Laboratories). Double stains were processed according to manufacturer's recommendations using the Rat and Mouse Double Stain Kit (Biocare Medical, Concord, CA). Stained slides were visualized with a Nikon Eclipse E400 microscope (Nikon Instruments Inc., Melville, NY). Images were captured with a SPOT Flex Shifting Pixel Color Mosaic (FX1520) digital camera, and were analyzed using the SPOT basic software package (Diagnostic Instruments Inc., Sterling Heights, MI). Images were processed for publication using Adobe Photoshop Elements 5.0 (Adobe Systems, San Jose, CA).

### Analysis of Mouse Gene Expression Microarrays

Total RNA was extracted using RNeasy (Qiagen, Germantown, MD). RNA integrity was assessed using an Agilent 2100 Bioanalyzer. All samples demonstrated RNA integrity (RIN) of 7.7 or greater. RNA was labeled using TotalPrep RNA (Ambion), and hybridized to Illumina MouseWG-6 v2 BeadChips for analysis of 45,281transcripts covering 30,774 genes. Data was interpreted using BeadStudio and quantile normalized to adjust for sample-to-sample variation. Differential analysis was conducted on a subset of 21,649 probes that were detected in at least one sample (detection *p*-value <0.01). Significance Analysis of Microarray (SAM) software [40] was used to determine differential expression with a false discovery rate (FDR) <1% and a minimum fold-change of 2 unless otherwise stated. Heatmaps were generated in R [41] using the heatmap.2 package. Probe expression data was Z-score normalized and hierarchical clustering was calculated on a Euclidean distance dissimilarity metric with an average clustering algorithm for heatmaps showing row and column dendrograms. Probes were displayed in descending order by fold-change in *Pten* +/− tumors. Gene microarray data is MIAME compliant and the raw data has been deposited in the MIAME compliant GEO database: accession # GSE19059 (NCBI tracking system #15737926).

### Quantitative real-time, RT-PCR Analysis

Total cellular RNA was extracted and processed into cDNA [39]. PCR reactions containing cDNA, Syber Green PCR Master Mix (Applied Biosystems, Inc.) and primers for mouse *Wnt3, Wnt7a, Vegfa,* and *Gapdh* were performed for 40 cycles in triplicate. Primer sequences are available upon request. Gene expression was normalized internally to *Gapdh* expression, accounting for differences in primer efficiencies. Results from at least three separate experiments were analyzed.

### Tissue Microarray

Construction of the tissue microarray (TMA) was performed at the National Institutes of Health consisting of 144 previously untreated paraffin-embedded childhood medulloblastoma tumor tissue specimens, obtained from the pathology libraries of Children's National Medical Center (CNMC) and the Armed Forces Institute of Pathology (AFIP). IRB approval was obtained from each institution for this TMA construction and analysis. The use of the specimens for construction of the TMA and subsequent biomarker molecular pathway analysis was approved by the Children's National Medical Center IRB and the Armed Forces Institute of Pathology IRB. Since these are from already existing diagnostic pathologic de-identified tissues, not obtained research specimens, the TMA specimens are considered non-human subjects research and are thus exempt from consent.

In brief, paraffin-embedded tissue samples were extracted using 0.6 µm diameter needles and mechanically embedded in a paraffin block into an array. Each sample was spotted into the block twice. 5 micron-thick sections were mounted onto positively charged slides for immunohistochemical analysis. A database relating each tumor specimen to its clinical and histologic characteristics was constructed and used for all correlations to the immunostainings. Standard IHC for PTEN (#9559, Cell Signaling) was performed. Immunostaining for PTEN was first performed using normal brain and classic medulloblastoma tissue samples to serve as negative control and for optimization of the staining procedure, respectively, prior to using the TMA for IHC. Each tissue sample in the array was independently scored for positivity by two neuropathologists (M.S. and E.J.R.). Scoring was performed blinded. Definitions for scoring of PTEN staining (score = 0–2) were established by the neuropathologists and were based on the relative diffuse cellular homogeneity observed with the specific target staining for each specimen tested. Of the 144 MB specimens, 33 were designated “not-amenable” for analysis to indicate that in the opinion of the grading neuropathologist, that either the IHC staining or the tissue integrity on the TMA was not of suitable quality to confidently grade the staining.

## Supporting Information

Figure S1 *SmoA1* +; *Pten* +/− mouse medulloblastomas up-regulate expression of *Wnt3* and *Wnt7a*. Analysis of gene expression in mouse medulloblastomas revealed higher expression of the Sonic Hedgehog pathway genes *Wnt3* and *Wnt7a* in tumors from *SmoA1* +; *Pten* +/− (white bars) versus from *SmoA1* +; *Pten* +/+ (back bars) mice. (A) Using real-time, RT-PCR we confirmed up-regulated expression of mRNA for downstream targets of the Sonic Hedgehog signaling pathway, *Wnt3* and *Wnt7a*. Relative expression of *Wnt3* and *Wnt7a* was increased 5.9 and 6.5-fold (*, p<0.05 for all transcripts), respectively in *SmoA1* +; *Pten* +/− (n = 7) medulloblastomas. *Error bars*, standard error of the mean. (B) This increased RNA expression correlated with a significant increase in expression of Wnt-3 and Wnt-7 protein by western blotting in *SmoA1* +; *Pten* +/− (n = 4), compared to *SmoA1* +; *Pten* +/+ (n = 3) mouse medulloblastomas.
(8.44 MB TIF)

Figure S2 *SmoA1* +; *Pten* +/− mouse medulloblastomas express markers of neuronal differentiation. (A) Analysis of gene expression in mouse medulloblastomas revealed higher expression of genes involved in neuronal differentiation, such as *Bdnf*, *Neurl*, *Id4*, and *Neurod2* in tumors from *SmoA1* +; *Pten* +/− (n = 8) versus from *SmoA1* +; *Pten* +/+ (n = 4) mice. Red pixels in the heatmap visualization represent increased expression and green pixels represent decreased expression of mRNA transcripts for the listed gene probes. (B) Immunohistochemical analysis of paraffin-embedded sections of the two tumor types confirmed significant expression of the marker of neuronal differentiation, NeuN, in *SmoA1* +; *Pten* +/- tumors (n = 5), and no expression of NeuN in tumors from *SmoA1* +; *Pten* +/+ mice (n = 5), except in the expected location in the internal granule layer (IGL). (C) Double-staining of tumor sections revealed diffuse proliferation with scattered areas of staining for NeuN in *SmoA1* +; *Pten* +/+ medulloblastomas. *Pten* deficient tumors displayed larger islands of neuronal differentiation, surrounded by and distinct from PCNA positive, areas of proliferation. (D) Neither tumor type stained positive for the marker of astrocytic differentiation, GFAP. (E) Staining for synaptophysin, a marker of primitive neurons was weak in both tumor types, but appeared to overlap with staining for PCNA.
(10.16 MB TIF)

Table S1 Survival of *Pten* +/+ versus *Pten* +/− mice.
(0.03 MB DOC)

Table S2 Comparison of medulloblastomas from *Pten* wild-type versus deficient mice.
(0.03 MB DOC)

Table S3 Differentially expressed genes in *SmoA1* +; *Pten* +/− mouse medulloblastomas.
(0.10 MB DOC)
