# Supplementary figures and images for: Heterozygosity for Pten Promotes Tumorigenesis in a Mouse Model of Medulloblastoma
Source: PLoS One. 2010 May 26;5(5):e10849. doi: 10.1371/journal.pone.0010849 (PMC2877103; doi:10.1371/journal.pone.0010849)

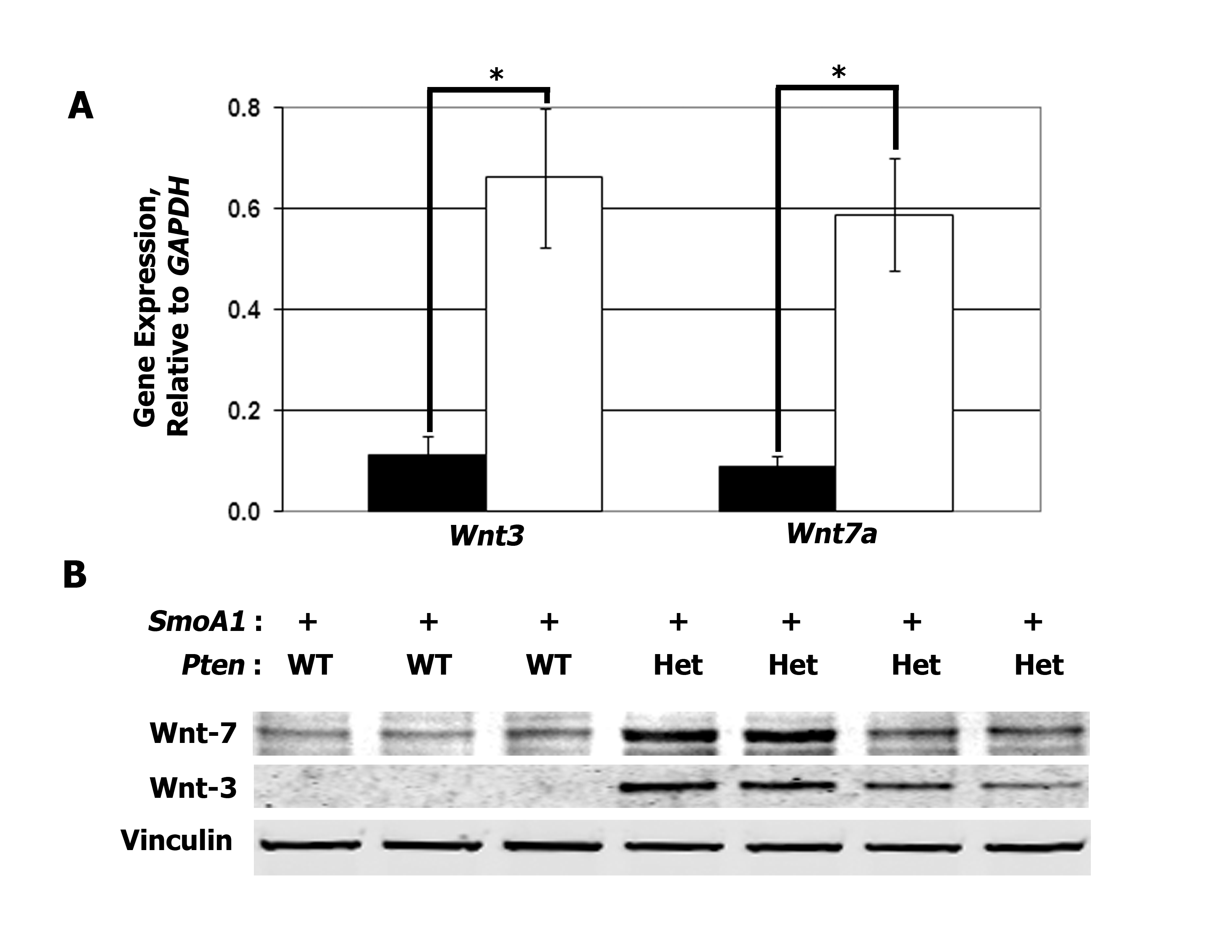

Supplement: Figure S1 — SmoA1 +; Pten +/− mouse medulloblastomas up-regulate expression of Wnt3 and Wnt7a. Analysis of gene expression in mouse medulloblastomas revealed higher expression of the Sonic Hedgehog pathway genes Wnt3 and Wnt7a in tumors from SmoA1 +; Pten +/− (white bars) versus from SmoA1 +; Pten +/+ (back bars) mice. (A) Using real-time, RT-PCR we confirmed up-regulated expression of mRNA for downstream targets of the Sonic Hedgehog signaling pathway, Wnt3 and Wnt7a. Relative expression of Wnt3 and Wnt7a was increased 5.9 and 6.5-fold (*, p<0.05 for all transcripts), respectively in SmoA1 +; Pten +/− (n = 7) medulloblastomas. Error bars, standard error of the mean. (B) This increased RNA expression correlated with a significant increase in expression of Wnt-3 and Wnt-7 protein by western blotting in SmoA1 +; Pten +/− (n = 4), compared to SmoA1 +; Pten +/+ (n = 3) mouse medulloblastomas. (8.44 MB TIF) [file pone.0010849.s001.tif]

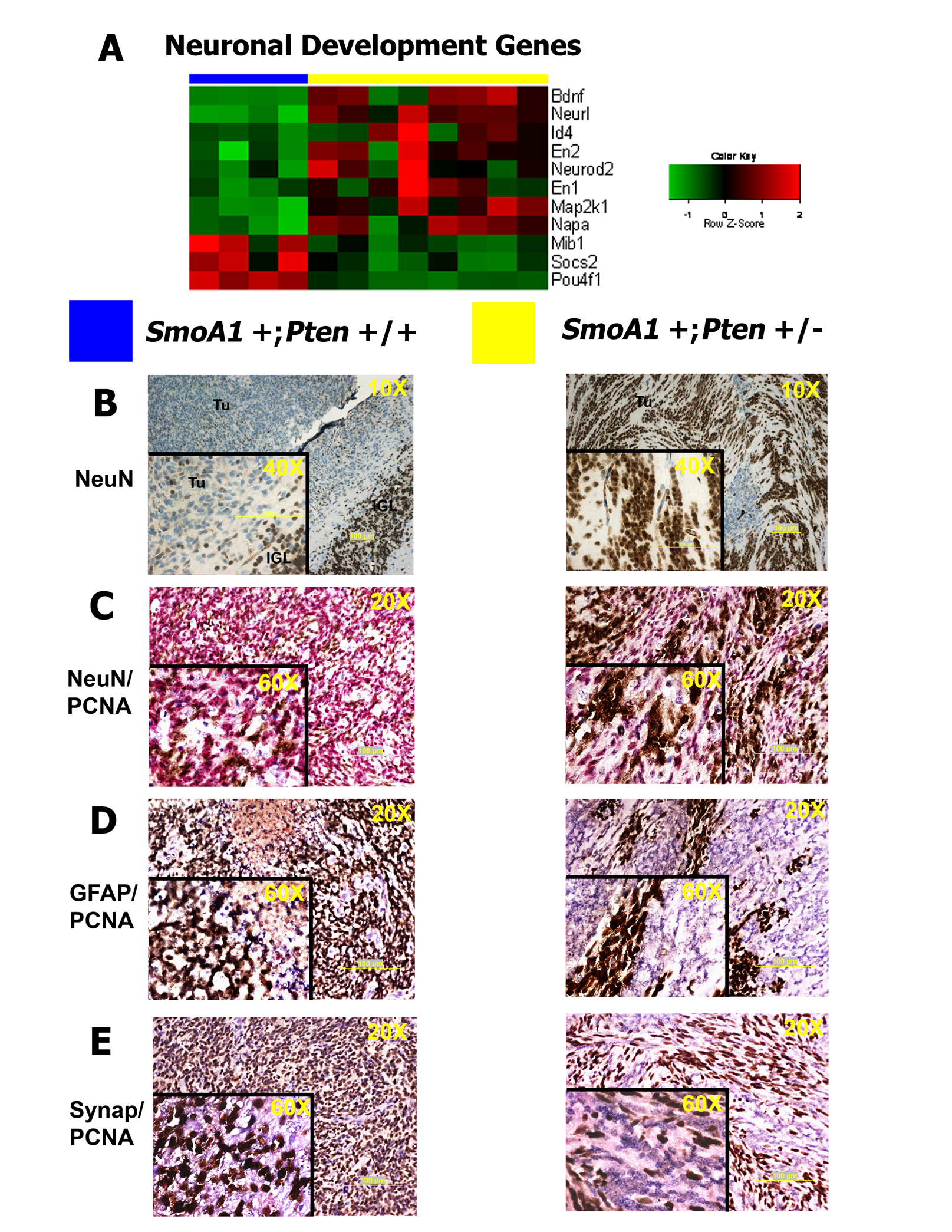

Supplement: Figure S2 — SmoA1 +; Pten +/− mouse medulloblastomas express markers of neuronal differentiation. (A) Analysis of gene expression in mouse medulloblastomas revealed higher expression of genes involved in neuronal differentiation, such as Bdnf, Neurl, Id4, and Neurod2 in tumors from SmoA1 +; Pten +/− (n = 8) versus from SmoA1 +; Pten +/+ (n = 4) mice. Red pixels in the heatmap visualization represent increased expression and green pixels represent decreased expression of mRNA transcripts for the listed gene probes. (B) Immunohistochemical analysis of paraffin-embedded sections of the two tumor types confirmed significant expression of the marker of neuronal differentiation, NeuN, in SmoA1 +; Pten +/- tumors (n = 5), and no expression of NeuN in tumors from SmoA1 +; Pten +/+ mice (n = 5), except in the expected location in the internal granule layer (IGL). (C) Double-staining of tumor sections revealed diffuse proliferation with scattered areas of staining for NeuN in SmoA1 +; Pten +/+ medulloblastomas. Pten deficient tumors displayed larger islands of neuronal differentiation, surrounded by and distinct from PCNA positive, areas of proliferation. (D) Neither tumor type stained positive for the marker of astrocytic differentiation, GFAP. (E) Staining for synaptophysin, a marker of primitive neurons was weak in both tumor types, but appeared to overlap with staining for PCNA. (10.16 MB TIF) [file pone.0010849.s002.tif]
