# Supplementary material for: Heterozygosity for Pten Promotes Tumorigenesis in a Mouse Model of Medulloblastoma
Source: PLoS One. 2010 May 26;5(5):e10849. doi: 10.1371/journal.pone.0010849 (PMC2877103; doi:10.1371/journal.pone.0010849)
Supplement: Table S1 — Survival of Pten +/+ versus Pten +/− mice. (0.03 MB DOC) [file pone.0010849.s003.doc]

**Table S1.**

**Survival of *Pten* +/+ versus *Pten*** +/- mice

| ***SmoA1*** | ***Pten*** | **Animals** | **Mortality (%)** |
| --- | --- | --- | --- |
| **+/+** | **+/+** | 100 | 75 |
| **+/-** | **+/+** | 54 | 50 |
| **+/-** | **+/-** | 60 | 98 |
