# Supplementary material for: Heterozygosity for Pten Promotes Tumorigenesis in a Mouse Model of Medulloblastoma
Source: PLoS One. 2010 May 26;5(5):e10849. doi: 10.1371/journal.pone.0010849 (PMC2877103; doi:10.1371/journal.pone.0010849)
Supplement: Table S2 — Comparison of medulloblastomas from Pten wild-type versus deficient mice. (0.03 MB DOC) [file pone.0010849.s004.doc]

**Table S2.**

**Comparison of medulloblastomas from *Pten* wild-type versus deficient mice**

| ***SmoA1*** | **+** | **+** |
| --- | --- | --- |
| ***Pten*** | **Wild-type** | **Deficient** |
| **Tumor Location** | Cerebellum | Cerebellum |
| **Cell Morphology** | Dense sheets of small, round blue cells | Large areas with extensive nodularity |
| **Tumor Cellularity** | Dense | Reduced |
| **Neuronal Differentiation (NeuN IHC)** | - | + |
| **Mitotic Figures** | Numerous | Rare |
| **Nuclear Karyorrhexis** | Numerous | Rare |
| **Proliferation (PCNA IHC)** | Diffuse; strong | Patchy; weak |
| **Cell Death (Cleaved Caspase 3 IHC)** | Scattered | Absent |

Abbreviations: *SmoA1*, mutated, constitutively activated *Smoothened*; *Pten*, *Phosphatase and tensin homolog*; NeuN, neuronal nuclei; PCNA, proliferating cell nuclear antigen; IHC, immunohistochemistry
