# Supplementary material for: Heterozygosity for Pten Promotes Tumorigenesis in a Mouse Model of Medulloblastoma
Source: PLoS One. 2010 May 26;5(5):e10849. doi: 10.1371/journal.pone.0010849 (PMC2877103; doi:10.1371/journal.pone.0010849)
Supplement: Table S3 — Differentially expressed genes in SmoA1 +; Pten +/− mouse medulloblastomas. (0.10 MB DOC) [file pone.0010849.s005.doc]

**Table S3.**

**Differentially expressed genes in *SmoA1* +; *Pten*** +/- mouse medulloblastomas

| **Pathway/Function** | **Gene** | **Symbol & Aliases** | **Fold Change** | **Function** |
| --- | --- | --- | --- | --- |
| **Phosphoinositide 3-Kinase (PI3K)** | *Diacylglycerol kinase, gamma* | *Dgkg, 90kDa* | ↑ 5.8 | Activation of protein kinase C and Ras guanyl nucleotide-releasing proteins |
|  | *Inositol polyphosphate-4-phosphatase, type II* | *Inpp4b* | ↑ 5.0 | Inositol phosphate-mediated signaling |
|  | *CDP-diacylglycerol synthase 1* | *Cds1* | ↑ 5.0 | CDP-diacylglycerol biosynthesis; phospholipid biosynthesis |
|  | *Phosphatidylinositol-4-phosphate 5-kinase, type 1 beta* | *Pip5k1b, Pipk5a/b; Pip5k1a; STM7* | ↑ 3.8 | Phosphatidylinositol metabolism |
|  | *Inositol 1,3,4-triphosphate 5/6 kinase* | *Itpk1* | ↑ 3.6 | Reversible, poly-specific inositol phosphate kinase |
|  | *Phosphatidylinositol-4-phosphate 5-kinase, type 1 gamma* | *Pip5k1c* | ↑ 3.1 | Type I phosphatidylinositol-4-phosphate 5-kinase localized in synapses and focal adhesion plaques |
|  | *Phosphatidylinositol 3 kinase, regulatory subunit, polypeptide 3* | *Pik3r3, p55pik* | ↑ 2.8 | Insulin receptor signaling pathway; intracellular signaling cascade |
|  | *CDP-diacylglycerol synthase 2* | *Cds2* | ↑ 2.3 | Regulates phosphatidic acid to CDP-diacylglycerol conversion, functions downstream of G protein-coupled receptors and tyrosine kinases |
|  | *Phospholipase C, beta 4* | *Plcb4* | ↑ 2.1 | Catalyzes the formation of inositol 1,4,5-trisphosphate and diacylglycerol from phosphatidylinositol 4,5-bisphosphate |
|  | *Phosphoinositide-3-kinase, catalytic, beta polypeptide* | *Pik3cb* | ↑ 1.9 | Component of 110-kD catalytic subunit that phosphorylates the 3-prime OH of inositol lipids |
|  | *Mitogen-activated protein kinase 8* | *Mapk8* | ↑ 1.5 | Key intermediate in multiple apoptotic pathways including TNF-alpha |
|  | *FK506 binding protein 12-rapamycin associated protein 1* | *Frap1* | ↑ 1.2 | Phosphatidylinositol kinase-related kinase mediates response to stress |
|  | *Phosphatase and tensin homolog* | *Pten* | ↓ 1.1 | Tumor suppressor, dephosphorylates phosphoinositide substrates, suppresses PI3K/AKT pathways |
| **VEGF / Angiogenesis** | *Tumor necrosis factor receptor superfamily, member 12a* | *Tnfrsf12a, HPIP; Fn14; TweakR* | ↑ 5.2 | Angiogenesis; apoptosis; cell adhesion; axon extension; development |
|  | *Secretogranin II* | *Scg2, SgII; Chgc* | ↑ 3.1 | Angiogenesis; cell motility; negative regulation of endothelial cell proliferation; chemotaxis; MAPKKK; inflammatory response |
|  | *Reticulon 4 (Rtn4), transcript variant 1* | *Rtn4; ASY; NSP-CL* | ↑ 3.1 | Angiogenesis; regulation of cell migration; inhibitor of axon extension |
|  | *Thymus cell antigen 1, theta* | *Thy1, CD90* | ↑ 3.1 | Angiogenesis; negative regulation of axonogenesis; cell-cell adhesion; negative regulation of apoptosis; mast cell activation |
|  | *Annexin A2* | *Anxa2, Cal1h* | ↑ 2.5 | Angiogenesis; collagen fibril organization; fibrinolysis |
|  | *Vascular endothelial growth factor A, transcript variant 1* | *Vegfa, VPF; VEGF-A; Vegf* | ↑ 2.3 | Angiogenesis; mesoderm development; epithelial cell differentiation; anti-apoptosis; blood vessel; cell migration |
|  | *FMS-like tyrosine kinase 1* | *Flt1, VEGFR1; Flt-1; sFlt1; VEGFR-1* | ↑ 2.1 | Angiogenesis; patterning of blood vessels; cell adhesion; cell differentiation; cell migration |
|  | *Neurotrophic tyrosine kinase, receptor, type 2* | *Ntrk2, trkB; Tkrb* | ↑ 2.0 | Vasculogenesis; mechanoreceptor differentiation; cell differentiation; development |
|  | *Heparin-binding EGF-like growth factor* | *Hbegf* | ↑ 1.8 | ErbB4 ligand |
|  | *Stabilin 1* | *Stab1, MS-1; STAB-1* | ↓ 2.6 | Negative regulation of angiogenesis; cell adhesion; inflammatory response |
| **Sonic Hedgehog (Shh)** | *Sonic hedgehog* | *Shh* | ↑ 1.3 | Key inductive signal in patterning of the ventral neural tube; cell surface localization |
|  | *GLI-Kruppel family member GLI2* | *Gli2* | ↓ 4.2 | Development including neuron development; proliferation |
|  | *Smoothened* | *Smo; Smoh* | ↓ 3.2 | Vasculogenesis; smoothened signaling pathway; central nervous system development |
|  | *Suppressor of fused* | *Sufu* | ↓ 2.9 | Suppressor of Hedgehog signaling |
|  | *Patched* | *Ptch1* | ↓ 2.7 | Smoothened signaling pathway; dorsal/ventral pattern formation |
|  | *Wingless-related MMTV integration site 4* | *Wnt4* | ↓ 2.4 | Wnt receptor signaling pathway; cell differentiation; development |
|  | *Hedgehog-interacting protein* | *Hhip; Hhip1; Hip1* | ↓ 2.3 | Smoothened signaling pathway; neuroblast proliferation; regulation of fibroblast growth factor receptor signaling pathway |
| **Cell Cycle** | *CDKN1A cyclin-dependent kinase inhibitor 1A* | *Cdkn1a, p21, Cip1, p21Waf* | ↑ 2.6 | Potent cyclin-dependent kinase inhibitor that binds and inhibits CDK2 or CDK4 thereby functioning as a regulator of cell cycle progression at G1; regulated by p53; interacts with proliferating cell nuclear antigen (PCNA) |
|  | *Cyclin-dependent kinase inhibitor 2B* | *Cdkn2b, p15, p15INK4A* | ↑ 2.3 | Cyclin-dependent kinase inhibitor; complexes CDK4 or CDK6 and prevents activation; cell growth regulator controlling G1 cell cycle progression; induced by TGF beta |
|  | *Cyclin-dependent kinase 6* | *Cdk6, PLSTIRE* | ↓ 3.7 | Protein kinase catalytic subunit important for G1 phase progression and G1/S transition. Phosphorylates tumor suppressor protein Rb |
|  | *Cyclin-dependent kinase 4* | *Cdk4* | ↓ 3.3 | Ser/Thr protein kinase catalytic subunit important G1 phase progression. Phosphorylates Rb |
|  | *Transformation related protein 53* | *Trp53* | ↓ 2.5 | Stress-induced regulator of cell cycle arrest, apoptosis, senescence, DNA repair, or changes in metabolism; functions as a tumor suppressor |
|  | *E2F transcription factor 1* | *E2f1* | ↓ 2.0 | Pivotal to cell cycle regulation; target of small DNA tumor viruses; preferentially binds Rb1 in a cell-cycle dependent manner |
|  | *Breast cancer 2* | *Brca2* | ↓ 1.9 | Germ line mutations in Brca2 confer risk to breast or ovarian cancer; maintainer of genome stability and homologous recombination pathway for double-strand DNA repair |
|  | *Ataxia telangiectasia mutated* | *Atm* | ↓ 1.8 | PI3/PI4-kinase family; cell cycle checkpoint kinase; regulates p53, BRCA1, CHK2, RAD17, RAD9, and DNA repair protein NBS1 |
|  | *Retinoblastoma 1* | *Rb1, pRB* | ↓ 1.8 | Negative regulator of cell cycle; stabilizes constitutive heterochromatin; hypophosphorylated form binds transcription factor E2F1 |
